# Detection of natural infection with *Mycobacterium intracellulare *in healthy wild-caught Chacma baboons (*Papio ursinus*) by ESAT-6 and CFP-10 IFN-γ ELISPOT tests following a tuberculosis outbreak

**DOI:** 10.1186/1471-2180-8-27

**Published:** 2008-02-07

**Authors:** Gerald K Chege, Robin M Warren, Nico C Gey van Pittius, Wendy A Burgers, Robert J Wilkinson, Enid G Shephard, Anna-Lise Williamson

**Affiliations:** 1 Department of Clinical Laboratory Sciences, Faculty of Health Sciences, University of Cape Town, Anzio Road, Observatory 7925, Cape Town, South Africa; 2 Division of Molecular Biology and Human Genetics, Department of Biomedical Sciences, DST/NRF Centre of Excellence in Biomedical Tuberculosis Research, US/MRC Centre for Molecular and Cellular Biology, Faculty of Health Sciences, Stellenbosch University, PO Box 19063 Tygerberg 7505, South Africa; 3 Department of Medicine, Faculty of Health Sciences, University of Cape Town, Anzio Road, Observatory 7925, Cape Town, South Africa; 4 Institute of Infectious Diseases and Molecular Medicine, University of Cape Town, Observatory 7925, Cape Town, South Africa; 5 Wellcome Trust Centre for Research in Clinical Tropical Medicine, Division of Medicine, Wright Fleming Institute, Imperial College London W2 1PG, UK; 6 MRC/UCT Liver Research Centre, Department of Medicine, Faculty of Health Sciences, University of Cape Town, Anzio Road, Observatory 7925, Cape Town, South Africa; 7 National Health Laboratory Service, Groote Schuur Hospital, Anzio Road, Observatory 7925, Cape Town, South Africa

## Abstract

**Background:**

Both tuberculous and non-tuberculous mycobacteria can cause infection in nonhuman primates (NHP), indicating the existence of potential zoonotic transmission between these animals and visitors to zoos or animal handlers in primate facilities. Screening of mycobacterial infections in NHP is traditionally done by tuberculin skin test (TST), which is unable to distinguish between pathogenic and non-pathogenic mycobacterial infections. In this study, we investigated the use of ESAT-6 and CFP-10 for detection of mycobacterial infections in a wild-caught baboon colony after one baboon died of tuberculosis (TB).

**Methods:**

Peripheral blood lymphocytes for interferon-gamma enzyme-linked immunospot assay (IFN-γ ELISPOT) assay were obtained from TST positive baboons and those in contact with tuberculous baboons before being euthanased, autopsied and lung tissues taken for histology and mycobacterial culture.

**Results:**

Both ESAT-6 and CFP-10 IFN-γ ELISPOT assays were able to detect early *M. tuberculosis *but also *M. intracellulare *infection. Although this indicates potential cross-reactivity with *M. intracellulare *antigens, the method was able to distinguish *M. bovis *BCG vaccination from *M. tuberculosis *infection. This assay performed better than the TST, which failed to detect one *M. tuberculosis *and two early *M. intracellulare *infections.

**Conclusion:**

These results suggest that the IFN-γ ELISPOT assay could improve the detection of *M tuberculosis *infections when screening NHP. There is some doubt, however, concerning specificity, as the assay scored positive three animals infected with *M. intracellulare*.

## Background

The majority of the disease-causing species of the genus *Mycobacterium *belong to two groups, the *M. tuberculosis *complex (MTC) and the *M. avium-intracellulare *complex (MAC). MTC comprises of *M. tuberculosis*, *M. bovis*, *M africanum *and the attenuated *M. bovis *bacille Calmette-Guerin (BCG). With the exception of BCG, these species are pathogenic and can cause tuberculosis (TB) in humans and animals [1-3]. Disease caused by MTC pathogens is characterised by tubercle formation in the infected tissues. These bacteria are considered obligate intracellular pathogens and the most efficient mode of transmission is via respiratory route [4]. In contrast, environmental mycobacteria have the capacity to survive and multiply under a wide range of environmental conditions [5] and to interact with a variety of environmental reservoirs, including protozoa and insects [6]. The majority of environmental mycobacteria are considered non-pathogenic. However, members of MAC are capable of causing nontuberculous infections and disease in a wide range of animal species [5,6]. MAC comprises of two species, *M. avium *and *M. intracellulare*, and although these species are considered opportunistic pathogens, their role in human and animal diseases and their pathogenic potential have become increasingly recognised, especially in immunocompromised persons with HIV-1 infection [7,8].

Non-human primates (NHP) are susceptible to infections caused by members of MTC [9-13] and MAC [14,15], including *M. intracellulare *[16,17], indicating the existence of potential zoonotic transmission between NHP and visitors to zoos and game parks as well as animal handlers and laboratory workers using biological products from NHP. The tuberculin skin test (TST) is the traditional *in vivo *screening test for detecting TB in NHP [18]. This test utilizes PPDs, which are crude antigen preparations, usually made from *M. tuberculosis*, *M. bovis *or *M. avium*. As PPD contains cross-reactive antigens [19], a positive TST could indicate tuberculous and non-tuberculous disease, prior vaccination with BCG, or immune sensitisation with non-pathogenic environmental mycobacteria. In addition, BCG vaccine vectors are increasingly being used in NHP studies [20-23], making it important to distinguish such vaccinations from natural pathogenic mycobacterial infections.

The 6-kDa early secretory antigenic target (ESAT-6) and culture filtrate protein 10 (CFP-10) are well defined mycobacterial proteins [24-26]. Both are low molecular-weight proteins which are secreted in the culture filtrate by *M. tuberculosis *and other pathogenic mycobacteria, including *M. bovis *[27], following short-term axenic culture. These proteins are absent from the genomes of all BCG substrains and those of several non-pathogenic environmental mycobacteria including *M. avium avium *and *M. avium paratuberculosis *[24,28]. Both ESAT-6 and CFP-10 induce strong T-cell IFN-γ responses [27,29-31], prompting their proposed use as diagnostic markers for *M. tuberculosis *and *M. bovis *infections [25,32,33]. However, the presence of ESAT-6 and CFP-10 orthologues in other mycobacterial species, including non-pathogenic species such as *M. smegmatis *and *M. gastri *[34] and environmental species like *M. flavescens *[28] has raised some concerns regarding the potential use of these antigens as diagnostic markers [35]. Paradoxically, the presence of ESAT-6 and CFP-10 in other mycobacterial species does not seem to confound the detection of *M. tuberculosis*- and *M. bovis*-associated specific responses in clinical and epidemiological practice [29,30,32,36-38], although these studies were mostly performed in low-incidence settings and patients may not have had significant previous contact with non-tuberculous mycobacteria. A study by Arend *et al *[39] showed that when ESAT-6 and/or CFP-10 enzyme-linked immunosorbent assay (ELISA) and enzyme-linked immunospot assay (ELISPOT) were used to measure interferon-gamma production, most *M. kansasii*- or *M. marinum*-infected patients and several persons exposed to environmental mycobacteria responded to ESAT-6 and/or CFP-10. IFN-γ production by T cells from leprosy patients, TB patients and unexposed controls in response to the *M. leprae *homologue of CFP-10 also showed significant crossreactivity with CFP-10 of *M. tuberculosis *[40].

In the present study, we evaluated ESAT-6 and CFP-10 for use in the interferon-gamma (IFN-γ) ELISPOT assay for diagnosis of mycobacterial infections in baboons held in a research facility in South Africa after the death of one baboon due to TB.

## Methods

### Baboons

Ninety wild-caught Chacma baboons (*Papio ursinus*) were involved in this study. Of these, 34 baboons had been newly introduced into the colony from the wild, while the other 56, including the one that died of *M. tuberculosis *infection, had been kept in the colony for over 3 years. Of the 56 baboons, 10 had been sensitised to BCG by experimental inoculation, 2 to 3 years prior to the current study. Ethical approval for the experimentation on the baboons was obtained from the Animal Ethics Committee of the University of Cape Town.

### Tuberculin Skin test

Baboons were anaesthetised with ketamine hydrochloride (10 mg/kg body mass; intramuscular injection) and injected intradermally with 100 μL (1000 IU in saline) of bovine tuberculin PPD (Institute of Animal Science and Health, Lelystad, The Netherlands) in the upper palpebrum of the eyelid. Reaction to tuberculin was checked by visual observation at 24, 48 and 72 hours and interpreted as TST positive or negative. The TST positivity reactions comprised of erythema of palpebrum alone, various degrees of erythema with minimum swelling or slight swelling of palpebrum without erythema, and obvious swelling of palpebrum with drooping of the eyelid or swelling and/or necrosis of the palpebrum with eyelid closed. Bruise-extravasation of blood associated with injection of tuberculin or no detectable reaction on the palpebrum were interpreted as TST negative.

### IFN-γ ELISPOT assay

Peripheral blood mononuclear cells (PBMC) were isolated from heparinised blood by standard Ficoll-gradient centrifugation. Freshly isolated or cryo-preserved PBMC were used in a standard IFN-γ ELISPOT assay as previously described [41]. Briefly, ELISPOT plates (MultiScreen-IP, Millipore) were coated overnight at 4°C with purified anti-human IFN-γ monoclonal antibody (clone 1-D1K, Mabtech). PBMC were incubated for 24 h at 37°C in triplicate at 200,000 cells per well with PPD (4 μg/mL), ESAT-6 (4 μg/mL), or CFP-10 (4 μg/mL) as the stimulant in a total volume of 0.1 mL. Triplicate wells with cells and PHA (4 μg/mL) and culture medium alone, served as positive and background stimulation controls respectively. The average of triplicate counts of IFN-γ spot forming cells (SFC) was calculated for each stimulant and normalised to 10^6 ^PBMC to give IFN-γ SFC/10^6 ^PBMC. The results were reported as net IFN-γ SFC/10^6 ^PBMC after subtracting the background SFC/10^6 ^PBMC obtained in the absence of any stimulant (PBMC plus culture medium alone).

To determine if a response was positive, a cut-off value was established for PPD, ESAT-6 and CFP-10 using PBMC from 27 TST negative healthy baboons whose background response or response to ESAT-6 or CFP-10 was <50 IFN-γ SFC/10^6 ^PBMC. The cut-off value was defined as the mean net IFN-γ SFC/10^6 ^PBMC after subtracting the value of background reaction plus 3 standard deviations of the mean or twice the highest value of background reaction, whichever was greater. The mean net responses plus three standard deviations for PPD, ESAT-6 and CFP-10 were 8 + 35, 12 + 31 and 5 + 22 IFN-γ SFC/10^6 ^PBMC respectively while the highest value of background reactions were 43, 35 and 30. Thus, the cut-off values for PPD, ESAT-6 and CFP-10 were determined as 86, 70 and 60 IFN-γ SFC/10^6 ^PBMC, respectively. The mean background stimulation in the absence of any stimulant and in response to the PHA positive controls were 5 ± 4 and 1562 ± 697 SFC/10^6 ^PBMC respectively.

### Euthanasia and necropsy

Baboons with a positive TST were euthanased for necropsy using sodium pentobarbital (200 mg/kg body mass). In addition, baboons that were caged adjacent to those which were found to have tubercles at necropsy (here referred to as contact baboons) were also euthanased for necropsy. All the TST-positive baboons had been kept in the colony for over 3 years while 6 of 8 contact baboons were less than 3 months in the colony. Before euthanasia, blood for PBMC isolation was collected from these animals. Also, as part of ongoing research, cryo-preserved PBMC were available from some of these animals. Organs in the thoracic and abdominal cavities were examined macroscopically for the presence of tubercles, adhesions or enlargement and/or caseation of lymph nodes. Samples for histology, mycobacteria-specific staining and culture were taken from the lung tissue and/or bronchial lymph nodes (BLN).

### Histology, ZN test and *M. tuberculosis *immunoperoxidase staining

Tissues were fixed in 10% buffered formalin and sent to a veterinary pathologist (VetPath; Pretoria) for histological examination, Ziehl-Neelsen (ZN) and *M. tuberculosis *immunoperoxidase (MTIP) staining.

### Mycobacteria culture and identification

Fresh tissues from the lung and/or BLN collected at necropsy were cultured for mycobacterial culture, using standard methods in the TB research laboratory of the Stellenbosch University. Standard precautions were followed to limit cross-contamination. All cultures were genotyped by the IS*6110 *Restriction Fragment Length Polymorphism (RFLP) method [42]. Isolates were also typed according to the *Mycobacterium tuberculosis *complex typing method developed in our laboratory [43]. *Mycobacterium tuberculosis *complex negative samples were further subjected to a 5'-16S rRNA gene PCR-sequencing assay, which is able to identify and speciate *Mycobacterium spp*. [44,45]. Used in combination these methods would be able to identify superinfections between mycobacterial species of MTC and MAC.

## Results

### Tuberculin Skin test

**Figure 1:**
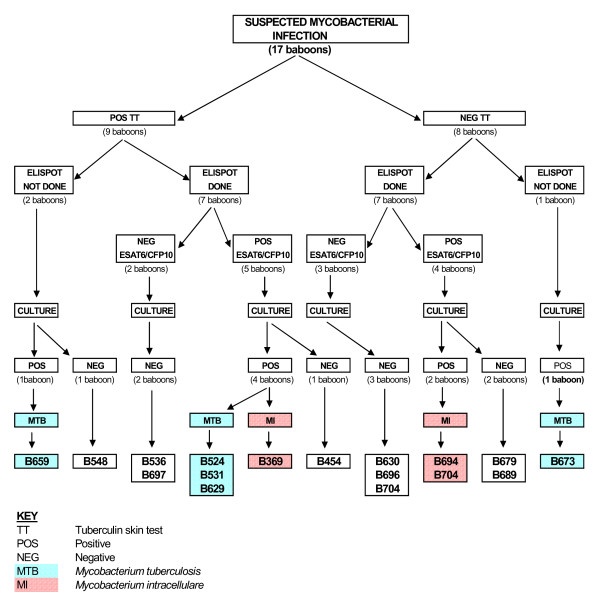
**Summary of the TST, ESAT-6 and CFP-10 IFN-γ ELISPOT assay and mycobacterial culture outcome**. A flow diagram showing the outcome of tuberculin skin test (TST), ESAT-6 and CFP-10 IFN-γ ELISPOT assay and mycobacterial culture.

Following the death of one baboon (B662) due to pulmonary TB diagnosed at necropsy, the remaining 89 baboons in the colony were screened for mycobacterial infection using bovine PPD tuberculin. Initially, only eight baboons tested positive, including three (B454, B536 & B548) with known prior vaccination with BCG or recombinant BCG. One additional baboon (B369) tested positive three months after initially testing negative, resulting in a total of nine tuberculin reactors (Figure 1 and Table 1). The test reaction ranged from severe (drooping of the eyelid, necrosis of the palpebrum with eyelid completely closed; B524, B531 & B629) to slight reactions (erythema of palpebrum with minimal swelling; B369, B454, B536 & B548). Reactions on eyelids of baboon B659 and B697 were moderate (obvious swelling of palpebrum without drooping or necrosis).

**Table 1:** Summary of findings

TST status	Baboon number	IFN-γ ELISPOT (SFC/10^6 ^PBMC)^#^			Pathology (lungs and BLN)		Special staining		Mycobacteria culture
			
		PPD	ESAT6	CFP10	Necropsy	Histopathology	ZN	MT IP	
**TST positive baboons**	B454Ψ	**902**	**774**	**157**	Negative	No granulomas; mild interstitial lymphocytic infiltration in BLN	+ (BLN only)	Negative	Negative
	B524	**2650**	**2163**	**2243**	Several tubercles; caseous LN	Multifocal necrogranulomas	Negative	+	*M. tuberculosis*
	B531	**210**	**89**	**109**	Several tubercles; caseous LN	Multifocal necrogranulomas; caseous necrosis in BLN	+	+	*M. tuberculosis*
	B536Ψ	**743**	20	11	Negative	No granulomas; mild peribronchial fibrosis;	+	Negative	Negative
	B548Ψ	ND	ND	ND	Negative	ND	ND	ND	Negative
	B629	**736**	**448**	**1348**	Several tubercles; caseous LN	Multifocal necrogranulomas	+	Negative	*M. tuberculosis*
	B659	ND	ND	ND	Single tubercle; single caseous LN	ND	ND	ND	*M. tuberculosis*
	B697	**1748**	0	0	A single tubercle-like lesion; enlarged hilar LN	Multifocal areas of granulomatous pneumonia	Negative	Negative	Negative
	B369*	**1397**	**1192**	**1170**	Negative	No granumomas; focal hyperplasia in BLN	Negative	Negative	*M. intracellulare*

**TST negative baboons**	B630	0	35	48	Negative	No granulomas; mild lymphatic hyperplasia in BLN	Negative	Negative	Negative
	B673	ND	ND	ND	Single caseous LN	Multifocal mild pneumonitis	+	+	*M. tuberculosis*
	B679	0	67	**109**	Negative	No granulomas; mild multifocal perivascular lymphocytic cuffing	Negative	Negative	Negative
	B689	78	**117**	**63**	Negative	Negative	Negative	Negative	Negative
	B694	0	**86**	**66**	Negative	Mild granulomatous inflammation	Negative	Negative	*M. intracellulare*
	B696	0	69	17	Negative	Negative	Negative	Negative	Negative
	B704	2	28	33	Negative	Negative	Negative	Negative	Negative
	B709	50	60	**138**	Negative	Negative	Negative	Negative	*M. intracellulare*

This table gives a summary of the major findings of the study. ND: not determined; BLN: bronchial lymph node; +: positive outcome; *: initially TST negative; Ψ: known prior vaccination with BCG or recombinant BCG; ^#^: cut-off values for PPD, ESAT-6 & CFP-10 = 86, 70, & 60 SFC/10^6 ^respectively (bold face indicate a positive response).

### Necropsy

The nine TST reactors and an additional eight contact baboons were euthanased for necropsy and further investigations. Of the nine TST positive baboons, four had classical tubercles in the lungs including at least one caseous BLN, one had a non-classical tubercle-like lesion (B697) with an enlarged but not caseous BLN and four had no macroscopic lesions (Table 1). Paradoxically, one of eight TST negative baboons (B673) had a single caseous BLN with no visible tubercles. The severity of lesions varied widely, ranging from numerous small tubercles in the lungs and one or more caseous BLN (B524, B531 and B629) to just a single caseous BLN (B673). These macroscopic lesions were restricted to the pulmonary system.

### Histology, ZN test and *M. tuberculosis *immunoperoxidase staining

As shown in the Table 1, necrotic granulomas and areas of necrosis were found in the lung and BLN tissues of three of nine TST positive baboons while other lesions such as lymphocytic infiltration and granulomatous inflammation, suggestive of mycobacterial infection, were found in four other TST reactors. Also, five of seven histologic lesions were confirmed by ZN and MTIP staining. For the TST negative baboons, only mild histological lesions were found in four of eight baboons and one (B673) was positive by ZN and MTIP staining.

### Mycobacterial culture and identification

Of the 17 baboons' specimens that were cultured, eight yielded mycobacterial growth. *M. tuberculosis *was identified in five and *M. intracellulare *in three of these cultures by PCR-based methods and 16S rRNA sequencing. *M. tuberculosis *was cultured from four of the nine TST reactors and one of eight TST negative baboons while *M. intracellulare *was cultured from one TST reactor and two TST negative baboons (Figure 1 and Table 1). It was unlikely that the culture of *M. intracellulare *represented laboratory cross-contamination as this species is rarely identified in sputum specimens routinely cultured in our laboratory. No superinfection was detected in any of these samples.

### IFN-γ ELISPOT assay

PBMC were isolated from some baboons at the time of TST or just before euthanasia for determination of IFN-γ ELISPOT response to bovine PPD, ESAT-6 and CFP-10. As shown in Figure 1 and Table 1, PPD ELISPOT test was positive for all TST positive reactors and negative for all TST non-reactors, indicating agreement between these two tests. However, two of the tuberculin reactors (B536 and B697) were negative for both ESAT-6 and CFP-10 ELISPOT test. The mycobacterial culture in these animals was also negative. The ESAT-6 and CFP-10 ELISPOT test was positive for B454, another TST reactor which had previously been vaccinated with BCG, although no mycobacteria was isolated from tissues from this animal. The ESAT-6 and CFP-10 ELISPOT test was also positive for B369, which was initially TST negative and mycobacteria culture positive for *M. intracellulare*. For the TST non-reactors, ESAT-6 and CFP-10 ELISPOT test was positive for four baboons, two (B694 and B709) from which *M. intracellulare *was isolated. However, no mycobacteria was isolated from the other two baboons (B679 and B689), which were also positive by ESAT-6 and CFP-10 ELISPOT.

**Figure 2:**
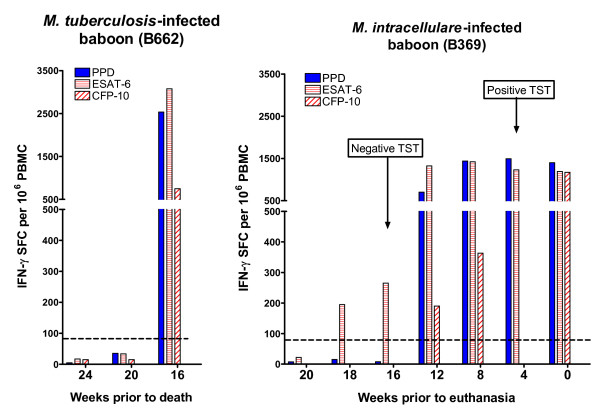
**IFN-γ ELISPOT responses to mycobacterial proteins at various times prior to death (B662) or euthanasia (B369)**. Three mycobacterial antigen preparations (bovine PPD, ESAT-6 and CFP-10) were used in IFN-γ ELISPOT assay using cryo-preserved PBMC from baboon B662 (infected with *M. tuberculosis*) and B369 (infected with *M. intracellulare*). The IFN-γ response to each protein was determined by subtracting the background (response to PBMC in the culture medium only) from the antigen response. Data points are the magnitudes of response to each antigen preparation at various time points. The dotted lines indicate the cut-off value for ESAT-6 (70 IFN-γ SFC/10^6 ^PBMC).

In an attempt to understand the kinetics of immune responses to *M. tuberculosis *and *M. intracellulare*, we performed retrospective measurements of T-cell IFN-γ responses in an ELISPOT assay using cryo-preserved PBMC, previously obtained from baboons B369 and B662. As depicted in Figure 2, the responses to ESAT-6 for B369 could be detected as early as 18 weeks before euthanasia but the PPD (and CFP-10) responses were detectable only 6 weeks later. Thus, the TST was initially negative at 16 weeks before euthanasia although the ELISPOT assay value was above the positive cut-off value for ESAT-6 (but not PPD and CFP-10). This delay in induction of a PPD response was not observed for B662 that died of TB.

## Discussion

The TST, which is a delayed-type hypersensitivity (DTH) reaction, is partially T cell-mediated [46] and IFN-γ plays a major role [47]. The major drawback of this test is the inability to distinguish active pathogenic infection from healthy carrier status, or from non-pathogenic exposure to environmental mycobacteria or BCG. The majority of TB outbreaks in NHP are associated with human contact or TB infected animals and carcasses [9,10,12] and rarely give rise to latent TB infection [11]. Thus, MTC infections in NHP are considered progressive and almost always fatal. However, infection with MAC is usually chronic [14-17] and sometimes refractory to TST [16]. In order to eliminate their zoonotic potential, early and reliable detection of potential pathogenic mycobacterial infections, including members of MAC in the nonhuman primate colonies is crucial.

Both ESAT-6 and CFP-10 have been proposed as diagnostic markers for pathogenic infection caused by *M. tuberculosis *and *M. bovis *in human and animals [25,31-33] because they are well defined antigens [24-26], absent from BCG vaccine strains and some non-pathogenic environmental mycobacteria [28,36], and they associate in *M. tuberculosis*-exposed people with a higher risk of developing active tuberculosis [29,30].

In our study, the IFN-γ ELISPOT response to ESAT-6, CFP10 and PPD were compared to the TST. There was concordance between the TST and PPD ELISPOT tests. However the results for ESAT-6 and CFP-10 ELISPOT and TST were discordant in two of seven TST-positive and four of seven TST-negative animals. One of the two TST-positive but ESAT-6 and CFP-10 negative baboon (B536) could be a result of previous exposure to BCG via inoculation with a recombinant BCG vaccine 2–3 years previously, but the other baboon (B697) possibly represents environmental sensitisation as this animal had been caught from the wild less than 3 months before the test and had no known exposure to BCG. This outcome demonstrates that ESAT-6 and CFP-10 ELISPOT test is capable of distinguishing BCG vaccination and environmental exposure by non-pathogenic mycobacterial infection (or TST false-positives) from TB disease. However, ESAT-6 and CFP-10 ELISPOT test for another TST-positive baboon (B454) that had previously been vaccinated with BCG, was not conclusive as no mycobacteria was isolated from tissues from this animal. It was noteworthy that one *M. intracellulare *infection (B369) was detected by both TST and ESAT-6 and CFP-10 ELISPOT tests. Since the methods used to identify and speciate the mycobacteria excluded the possibility of superinfection with *M. tuberculosis*, this result suggests a cross-reactivity between *M. tuberculosis*-derived ESAT-6 and CFP-10 antigens and *M. intracellulare*. Paradoxically, TST failed to detect two other *M. intracellulare*-infected baboons (B694 and B704), possibly because the stage of infection in these animals were too early for the PPD-specific T responses to have developed.

Our data on the kinetics of immune responses induced by *M. tuberculosis *and *M. intracellulare *show that induction of PPD responses occurs much later than that of ESAT-6 in case of *M. intracellulare *infection. Thus, it is possible for TST to miss early *M. intracellulare *infection, as we observed in this study. However, the isolation of *M. intracellulare *from two other TST-negative but ESAT-6 and CFP-10 ELISPOT-positive baboons (B694 and B709) casts some doubt regarding the specificity of this ELIPOT test. This needs to be resolved by further investigation using larger sample sizes.

Although limited by the small sample size and an assumed exposure in the in-contact baboons, the findings in this study indicate that the ESAT-6 and CFP-10 ELISPOT assays were highly effective in detecting *M. tuberculosis *and *M. intracellulare *infections (although the detection of *M. intracellulare *was unsuspected and ostensibly indicates cross-reactivity). Further, these results show the ability of this method to distinguish BCG vaccination from infection with these mycobacteria. In addition, our results show that the TST failed to detect two infections with *M. intracellulare *and one infection with *M. tuberculosis*. We further show that T-cell responses to ESAT-6 and CFP-10 develop much earlier than those of PPD, especially in *M. intracellulare *infection and hypothesise that this could possibly facilitate earlier diagnosis of mycobacterial infection by ELISPOT test.

## Conclusion

This study demonstrated that the traditional TST can manifest significant false-negatives in screening for tuberculosis in NHP while the ESAT-6 and CFP-10 ELISPOT assays are highly effective in detecting *M. tuberculosis *and *M. intracellulare *infections, thus facilitating an improvement in identifying and controlling a potential zoonotic transmission hazard, especially where NHP are infected with *M. intracellulare*. The study also presents the first evidence of cross-reactivity between *M. tuberculosis *ESAT-6 and CFP-10 and *M. intracellulare *antigens, where animals infected with *M. intracellulare *react to ESAT-6 and CFP-10 of *M. tuberculosis*. Whilst this study may support the use of an ESAT-6 or CFP-10 IFN-γ ELISPOT assay to screen for tuberculosis in NHP facilities, a positive result may arise from infection by mycobacteria other than *M. tuberculosis*.

## Competing interests

The author(s) declare that they have no competing interests.

## Authors' contributions

GKC participated in collection and subsequent delivery of animals' samples to the laboratory, processing PBMC and performing of the ELISPOT tests, helped in TST and autopsies, compiled data and drafted primarily the manuscript. RMW performed cultures, mycobacteria speciation and helped drafting the manuscript. NCGvP helped in mycobacteria speciation and drafting the manuscript. WAB helped in ELISPOT assay and compiling of data. RJW participated partially in necropsies and helped in drafting the manuscript. EGS contributed in ELISPOT assay analysis and facilitating histopathology. A-LW was the principal investigator and was responsible for the study design. All the authors read and approved the final manuscript.
